# ‘The photographic negative of pulmonary oedema’ in COVID-19 pneumonia

**DOI:** 10.1136/postgradmedj-2020-139265

**Published:** 2020-11-12

**Authors:** Navneet Arora, Mohan Kumar H

**Affiliations:** Internal Medicine, Post Graduate Institute of Medical Education and Research, Chandigarh, India; Internal Medicine, Post Graduate Institute of Medical Education and Research, Chandigarh, India

**Figure 1:**
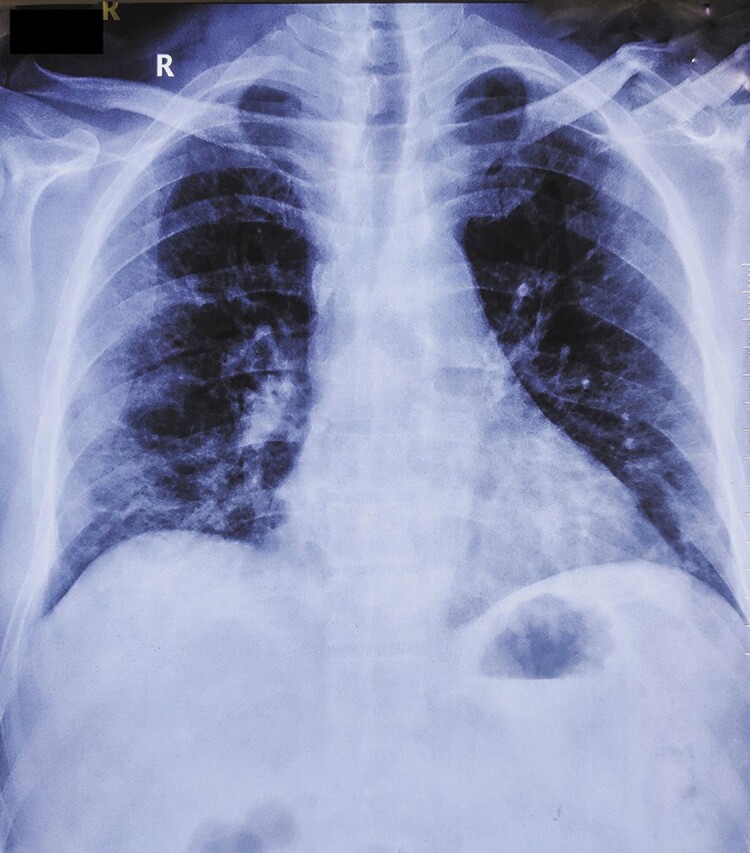
Chest X-ray showing bilateral peripheral opacities with central sparing giving appearance of reverse batwing.

A 53-year-old man presented to the emergency room (ER) with fever, dry cough and shortness of breath for 6 days. Clinically he had tachycardia (114 beats per minute), tachypnoea (30 per minute) and was maintaining oxygen saturation of 88% on room air. He had acute respiratory distress syndrome (ARDS) (PaO_2_/FiO_2_ of 0.28), and there was no leucopaenia or lymphopaenia. Chest X-ray revealed peripheral consolidations with base towards pleura and sparing of peri-hilar region consistent with a reverse batwing appearance (figure 1). The patient’s nasopharyngeal swab was tested for SARS Cov-2 RT-PCR, and it was positive. He was diagnosed to have COVID-19 pneumonia and started on oxygen supplementation and supportive care. The patient gradually improved and was discharged. In resource-constrained settings, a chest radiograph is the only investigation available for most patients. The findings have been used to support the diagnosis, determine the severity, guide the treatment and assess the treatment response. COVID-19 pneumonia causes peripheral consolidations, sparing the centre which gradually merges giving a reverse batwing appearance which is also known as ‘the photographic negative of pulmonary oedema’. The findings typically peak 10–12 days after the symptom onset.^1  2^ It is recommended that COVID-19 patients receive only chest X-ray as their primary means of imaging assessment. The sensitivity of Chest X-Ray in COVID-19 is estimated to be 69%.^3^
